# Biomaterials in Valvular Heart Diseases

**DOI:** 10.3389/fbioe.2020.529244

**Published:** 2020-12-09

**Authors:** Bita Taghizadeh, Laleh Ghavami, Hossein Derakhshankhah, Ehsan Zangene, Mahdieh Razmi, Mehdi Jaymand, Payam Zarrintaj, Nosratollah Zarghami, Mahmoud Reza Jaafari, Matin Moallem Shahri, Adrineh Moghaddasian, Lobat Tayebi, Zhila Izadi

**Affiliations:** ^1^Department of Medical Biotechnology, School of Advanced Medical Sciences, Tabriz University of Medical Sciences, Tabriz, Iran; ^2^Laboratory of Biophysics and Molecular Biology, Department of Biophysics, Institute of Biochemistry and Biophysics, University of Tehran, Tehran, Iran; ^3^Pharmaceutical Sciences Research Center, Health Institute, Kermanshah University of Medical Sciences, Kermanshah, Iran; ^4^Department of Bioinformatics, Institute of Biochemistry and Biophysics, University of Tehran, Tehran, Iran; ^5^Department of Biochemistry, Institute of Biochemistry and Biophysics, University of Tehran, Tehran, Iran; ^6^Nano Drug Delivery Research Center, Health Technology Institute, Kermanshah University of Medical Sciences, Kermanshah, Iran; ^7^Polymer Engineering Department, Faculty of Engineering, Urmia University, Urmia, Iran; ^8^Biotechnology Research Center, Pharmaceutical Technology Institute, Mashhad University of Medical Sciences, Mashhad, Iran; ^9^Department of Pharmaceutical Nanotechnology, School of Pharmacy, Mashhad University of Medical Sciences, Mashhad, Iran; ^10^Cardiology Department, Taleghani Trauma Center, Mashhad University of Medical Sciences, Mashhad, Iran; ^11^School of Medicine, Mashhad University of Medical Sciences, Mashhad, Iran; ^12^Marquette University School of Dentistry, Milwaukee, WI, United States; ^13^Department of Regenerative Medicine, Cell Science Research Center, Academic Center for Education, Culture and Research (ACECR), Royan Institute for Stem Cell Biology and Technology, Tehran, Iran

**Keywords:** valvular heart diseases, heart valve replacement, cardiac valve regeneration, tissue-engineered heart valves, biomaterials

## Abstract

Valvular heart disease (VHD) occurs as the result of valvular malfunction, which can greatly reduce patient’s quality of life and if left untreated may lead to death. Different treatment regiments are available for management of this defect, which can be helpful in reducing the symptoms. The global commitment to reduce VHD-related mortality rates has enhanced the need for new therapeutic approaches. During the past decade, development of innovative pharmacological and surgical approaches have dramatically improved the quality of life for VHD patients, yet the search for low cost, more effective, and less invasive approaches is ongoing. The gold standard approach for VHD management is to replace or repair the injured valvular tissue with natural or synthetic biomaterials. Application of these biomaterials for cardiac valve regeneration and repair holds a great promise for treatment of this type of heart disease. The focus of the present review is the current use of different types of biomaterials in treatment of valvular heart diseases.

## Introduction

According to the American Heart Association’s 2019 report, cardiovascular diseases remain a major source of mortality and disease-related cost burden in the United States and worldwide (Benjamin et al., 2019). Some of the major heart and circulatory diseases include valvular heart disease (VHD), coronary heart disease, aortic disease, myocardial infarction, cardiac myopathy, and heart failure. All these conditions involve physical damage to the heart resulting in functional loss.

In particular, VHD occurs as the result of valvular malfunction. Left untreated, valvular diseases can lead to stroke, heart failure and death due to sudden cardiac arrest (SCA). Different treatment regimens are available for management of VHD, including medications and lifestyle changes, as well as surgical and interventional procedures. Although most of these treatments can be helpful in reducing the symptoms, the gold standard approach is to repair or replace the injured valve tissue in order to restore full cardiac function. On the other hand, some impaired cardiac valves cannot be repaired (in 70% of cases) and need to be replaced by biomaterials (Yacoub and Cohn, 2004). Although cardiac regeneration ability immediately after birth has been indicated in humans, adult valvular tissues lack self-regeneration capacity following injuries, which can eventually lead to heart failure and death (Rabkin-Aikawa et al., 2005; Uygur and Lee, 2016). Various surgical valve repair and replacement procedures are available in order to avoid these fatal consequences.

As life expectancy and access to screening methods has improved across the world, the number of VHD cases has increased considerably in the past few years. Many people with valvular defects have no symptoms and in most cases, this condition remains constant throughout the person’s life. However, for other people, VHD slowly progresses and the symptoms become worse. Estimations suggest that the prevalence of VHD may increase up to 122% by the end of 2056, creating a significant socio-economic impact on the society (D’arcy et al., 2016). Currently, mitral regurgitation and aortic stenosis are the most common types of VHDs worldwide (Nkomo et al., 2006).

In the last decade, along with the tremendous growth in biomaterials applications, there have been numerous successful implementation of biomaterials in the replacement and repair of body tissues, including cardiac valves. Biomaterials are simply defined as materials that either would interact with body’s tissues for medical purposes, as implanted medical devices or used to support, augment or replace injured tissues and/or organs or as drug carriers. The main characteristics of a biomaterial substance include bio-functionality, biocompatibility, bioactivity, sterilizability and bio-inertia.

Various synthetic and natural biomaterials have been used for replacement and repair of damaged valvular tissues (Ratner, 2013). These biomaterials must be blood compatible and be able to interact with the physiological environment of cells or tissues in which they are being implanted. The most important parameters of a biomaterial that need to be assessed prior to implantation are physical properties, mechanical strength, geometry as well as friction, flow and wear resistance. Except the aforementioned materials, other types of biomaterials have also been studied and used for this purposes. In the present review, we will discuss the most commonly used biomaterials in treatment of heart valve diseases.

## Valve Heart Diseases (VHD)

Heart valve diseases are among the most common CVDs in the world, affecting nearly 2.5% of the US population (Brinkley and Gelfand, 2013). Potential risk factors related to VHD includes genetics, age, male gender, hypertension, hyperlipidemia, smoking, diabetes, and adrenal insufficiency, as well as rheumatic disease and infectious endocarditis. Despite challenges in identifying the underlying genetic basis of familial VHDs, mainly due to genetic and phenotypic heterogeneity of the disease, incomplete penetrance, and genetic modifiers, recent studies using genome-wide linkage analysis and transcriptomics approaches, transgenic animals, and microRNAs have been able to demonstrate genetic mechanisms of common VHDs, including bicuspid aortic valve (BAV) and mitral valve prolapse (MVP). Identifying these genetic cues can significantly improve treatment strategies for VHD patients and their at-risk family members (Lahaye et al., 2014; Helgadottir et al., 2018; Nagy et al., 2019; Chen et al., 2020).

There are two main classes of VHD—valvular stenosis and valvular insufficiency. Valvular stenosis occurs because of the stiffening and reduced elasticity of valve leaflets, which can lead to narrowing of the valve opening and subsequently, reduction of blood flow. Valvular insufficiency or regurgitation occurs as the result of incomplete closure of valve leaflets, leading to leakage or regurgitant flow development (Topilsky, 2018; Crousillat and Wood, 2019).

According to American Heart Association (AHA)/American College of Cardiology (ACC) guidelines, VHD progression can be categorized according to disease stage as follow (Nishimura et al., 2014):

*Stage A*: Patients who have the risk factors for development of VHD.
*Stage B*: Asymptomatic patients with progressive VHD (mild to moderate severity).
*Stage C*: Asymptomatic patients with severe VHD with either normal right or left ventricular systolic function (Stage C_1_) or decompensated ventricular function (Stage C_2_).
*Stage D*: Symptomatic patients with severe VHD.

VHD diagnosis might be challenging since patients may be asymptomatic or VHD might be accompanied by possible comorbidities. In this regard, precise patient evaluation in terms of family history and risk factors as well as careful physical examination (including intra- and extra-cardiac conditions and comorbidities) are mandatory.

VHD conditions can be congenital or acquired and may involve any of the four valves of the heart, however, when the aortic and mitral valves are involved, patient’s condition is often more serious. Age is another determining risk factor in VHD, in a way that the risk of VHD increases from less than 1% in people in the age of 18–44, to more than 13% in people over 75 years old (Brinkley and Gelfand, 2013).

Since the heart valve tissue cannot regenerate spontaneously, replacement (with biological or mechanical heart valves), repair (via reconstructive surgery) or interventional catheterization are the current treatment options for management of VHD. Abnormal valves that are not repairable are replaced with bio-prosthetic (i.e., biological) or mechanical (prosthetic) valves (Borer et al., 2004). Extensive research has been conducted on the design and development of biomaterials used in heart valves, with the aim of achieving the closest replica to natural human heart valves.

Generally, cardiac valve biomaterials should be biocompatible with good hemodynamics, high mechanical stability and resistance to degradation and calcification. Despite recent advances in valvular replacement, mechanical and bio-prosthetic heart valves, both share the disadvantage of remodeling and growth following implantation, which is considered a major limitation in their application—especially in children with congenital valve disorders, since the transplanted heart valve should be able to simultaneously grow along with the patient’s heart. Tissue-engineered heart valves (TEHVs) are possible substitutes for bio-prosthetic and mechanical valves, with the ability of remodeling and acquiring patient’s normal valve structure.

## Biomechanics of Heart Valves

Understanding the mechanical properties of natural heart valves is essential for successful design and fabrication of prosthetic valves so they can more closely resemble native valve’s structure and function. Cardiac tissue is composed of different cell types including cardiomyocytes, fibroblasts, endothelial, and smooth muscle cells. Fibroblasts are the most frequent cell types in the myocardium, which are responsible for the extracellular matrix (ECM) components deposition (Mackenna et al., 2000). Cardiac valve tissue is a mechanically active and a complex system of different cell types and their interactions with the components of ECM. The main valvular cell types are valvular interstitial cells (VICs) and valvular endothelial cells (VECs), which are responsible for synthesizing and maintaining the ECM, as well as maintaining the heart valve’s hemostasis, structure and integrity (Taylor et al., 2003). Cardiac ECM is a complex network of proteins involved in strength and elasticity—including collagen, elastin, fibronectin, glycosaminoglycans (GAGs), glycoproteins (GPs), and proteoglycans (PGs)—with collagen being the most common protein in the heart valve tissue (Schoen, 1997; Brand et al., 2006; Rienks et al., 2014). Excellent biomechanical features of heart valves is the result of close interactions between VECs and VICs, as well as their interactions with ECM, in response to biological and mechanical stimuli. In cellular level, VECs are the first valvular components on the leaflet’s surface that are exposed to hemodynamic shear stress of the blood flow. The biomechanical stimuli induced by shear forces result in gene expression alterations in these cells (Butcher et al., 2006). Valvular ECM functions as an organizing matrix for cellular support, which transmits biomechanical stimuli to the VICs, inducing subsequent VIC’s phenotypical changes as well as ECM remodeling (Rabkin-Aikawa et al., 2004). According to a study by El-Hamamsy and colleagues, the interactions between VICs and VECs seem to unify different components of valve tissue and brings mechanical integrity to the valve (El-Hamamsy et al., 2009).

Four cardiac valves provide unidirectional forward blood flow during each cardiac cycle. Two types of cardiac valves exist: semilunar valves (including pulmonary and aortic valves) with three identical leaflets or cusps and sigmoid or atrioventricular valves (including mitral and tricuspid valves). The sigmoid valves have a more complex structure compared to semilunar valves due to their asymmetrical geometry and unequal leaflet size (Sacks et al., 2009).

All the four heart valves lie on the basal plane of the heart, made from dense collagen fibers, which help maintain their constant position relative to the movements of other parts of the heart. While the mitral and tricuspid valves are responsible for regulating the blood inflow to the heart, the aortic and pulmonary valves control the blood flow from the left and right ventricles into the aorta and pulmonary artery, respectively (Hasan et al., 2014).

Each valvular leaflet is made of three different layers including fibrosa, spongiosa, and the ventricularis with their distinct ECM composition. All these layers are composed of VICs with their surrounding ECM, covered with VECs. Fibrosa is the main load bearing and the thickest layer of the valve leaflet, which is made of type I collagen fibers that can endure high tensile forces. Ventricularis is made of dense collagen and radially aligned elastin fibers and is responsible for reducing the radial strain during valve opening and maximum forward blood flow. The spongiosa layer provides lubrication during leaflet bending and pressurization and is composed of collagen fibers, hydrated PGs, and GAGs. Hydrated PGs and GAGs are also involved in oxygen and nutrient elements diffusion through the valve tissue, as well as growth factor sequestration and controlled release (Latif et al., 2005; Sacks et al., 2009; Li et al., 2019).

Heart valves undergo 30 million heart cycles a year, while operating under high dynamic and mechanical forces (fluid shear stress, hydrodynamics, flexural, and tensile forces) (Sacks et al., 2009; Butcher et al., 2011). These forces are induced and controlled by surrounding hemodynamic environment of the heart and are inflicted periodically; valve opening (flexural force), blood flow through the valve (shear stress and hemodynamic pressure), valve closure (flexural force) and prevention of blood back-flow (tensile forces). The trans-valvular pressure on each heart valve under physiological conditions is as follows: tricuspid valve = 25 mmHg, mitral valve = 120 mmHg, pulmonary valve = 10 mmHg, and aortic valve = 80 mmHg. The ability of heart valves to endure such mechanical forces throughout a person’s lifetime is due to their constant remodeling and adjusting their ECM.

Natural valve leaflets represent a dynamic biological material with a heterogeneous anisotropic structure and complex mechanical behavior. They present the optimum structure and geometry, which minimizes the shear stress resistance while maximizing the blood flow. Some of the most important features of heart valves include dynamic motion, complex surface geometry, anisotropic deformation, thin leaflets, and remodeling (Guyton and Hall, 1996; Sacks et al., 2006, 2009; Loerakker et al., 2013). The precise combination of all these multi-modal features ensure heart valve’s functionality throughout a person’s entire lifetime. Altogether, biomechanical valvular features are among determining factors that should be considered in biomaterial selection, as well as the design and engineering of bio-prosthetic or mechanical heart valves. According to CDC, 61% of VHD-related deaths are due to aortic valve diseases. Since aortic valve involvement leads to serious health complications, biomechanical behavior of healthy and abnormal aortic valve is particularly discussed in the following section.

The common aortic valve is a tricuspid valve responsible for maintaining the unidirectional blood flow from the left ventricle into the aorta. The leaflets does not have uniform thickness, their thickness increases toward free cusps margins (Ho, 2009). Similar to other heart valves, each aortic leaflet is composed of three layers (fibrosa, spongiosa, and ventricularis). Aortic valve stenosis is the most common aortic valve disease, which is caused by thickening and calcification of the cusps (Rajamannan et al., 2011). The peak velocity of the blood flow through aortic valve during each cardiac systole is approximately 1.35 m/s (Balachandran et al., 2011). When aortic leaflets become calcified, the blood flow velocity and the hemodynamic pressure on the aortic valve increases due to increased cusps thickness (velocity may exceed 4 m/s), which eventually leads to progression of valvular stenosis. The aortic valve also endures shear stress during cardiac systole (when blood flow passes the leaflets) and during diastole (when the blood pools into the sinuses). Valve closure induces flexural stress during diastole. As mentioned previously, biomechanical stimuli induce phenotypic and gene expression profile changes in valvular cells. Different hemodynamic forces are inflicted on the aortic and ventricular sides of the aortic valve during each cardiac cycle, which requires different mechanical features on each side of the cusps. The ventricular surface of aortic cusps are exposed to unidirectional shear stress while the aortic side is exposed to oscillatory shear stress (Sacks and Liao, 2019). This leads to side-dependent VEC biology, which means that VECs on different sides of the aortic leaflets have distinct phenotype and gene expression profiles. Based on previous reports, calcified aortic valve disease (CAVD) initiates from the aortic side of the leaflets while the spongiosa and ventricularis layers remain disease-protected (Yip and Simmons, 2011). Majority of patients suffering from aortic stenosis are presented with concomitant aortic regurgitation as well (Honda et al., 2012). Aortic regurgitation in these patients mainly occurs due to the loss of stretch in calcified cusps during diastole (Balachandran et al., 2011). A bicuspid aortic valve (BAV) with only two cusps instead of three is present in 1–2% of the population due to a congenital abnormality (Mordi and Tzemos, 2012). The normal aortic valve has circular ring whereas BAV has oval-shaped ring geometry. Oval geometry of BAV leads to high-frequency unsteady shear force on the cusps leading to early calcification and valve failure (Yap et al., 2012). Calcified valves in BAV, have reduced effective orifice area, which is associated with increased transvalvular pressure and subsequent elevated mechanical strain on the cusps (Chien et al., 2016). Major complications associated with BAV include aortopathy (thoracic aorta dilation) and aorta coarctation, both of which lead to early stenosis and valve failure. Due to severe complications associated with BAV, the ultimate treatment option for these patients is surgical. Surgical management of BAV patients is similar to those with tricuspid aortic valve disease.

## Application of Biomaterials in Heart Valves

### Prosthetic Heart Valves (PHVs)

PHVs or mechanical heart valves fabricated from synthetic materials (Table 1) have lifetime durability. However, patients who have received PHV transplants require lifetime consumption of anticoagulation medications, which can increase the chance of thromboembolism and hemorrhage (Russo et al., 2008; Chiang et al., 2014; Glaser et al., 2016). Synthetic materials are more advantageous compared to natural biomaterials, as their properties—such as degree of porosity, pore size, and 3D structure as well as mechanical strength and degradation time—can be controlled through the synthesis process (Kim and Mooney, 1998). However, issues related to their biocompatibility and subsequent inflammation, thrombosis or thromboembolism can cause limitations in their application. Moreover, prosthetic valve endocarditis (PVE) may also occur following prosthetic valve transplant, which may lead to surgical removal of the prosthesis (Wilson et al., 1982). Toxicity of synthetic materials, especially in the case of biodegradable materials, should also be considered.

**TABLE 1:** Commonly used natural and synthetic biomaterials in heart valve replacement and repair.

**Mechanical Heart valves**	**Biomaterials (Natural and Synthetic)**	**References**
	Polypropylene	Detmer et al., 1972; Gentle and Juden, 1984; Thornton et al., 1997
	Polyurethane urea	Jayabalan et al., 2000; Thomas et al., 2001; Thomas and Jayabalan, 2009
	Polyurethane (PU)	Mackay et al., 1996; Bernacca et al., 1997a,b, 2002b; Wheatley et al., 2000
	Polyvinyl alcohol (PVA)	Tadavarthy et al., 1975; Wan et al., 2002; Jiang et al., 2004; Millon and Wan, 2006
	Silicone	Carmen and Kahn, 1968; Carmen and Mutha, 1972; Cuddihy et al., 1976; Parfeev et al., 1983; Smith and Black, 1984
	Titanium	Mitamura et al., 1989; Jones et al., 2000; Aagaard, 2004; Leng et al., 2006; Guldner et al., 2011
	Pyrolytic carbon	Björk, 1972; Schoen et al., 1982; Goodman et al., 1996; Ritchie, 1996; Ely et al., 1998; Litzler et al., 2007
	Polyether urethane (PEU)	Boretos et al., 1975; Bernacca et al., 1995, 1997a; Wheatley et al., 2000
	Polycarbonate urethane (PCU)	Daebritz et al., 2004; Sachweh and Daebritz, 2006; Clauser et al., 2014
	Polyethylene glycol (PEG)	Paul et al., 1998; Ota et al., 2007; Ouyang et al., 2008; Zhou et al., 2013; Tseng et al., 2014; Zhang et al., 2015
Biological Heart valves	Gelatin	Tayama et al., 2000; Wong et al., 2010; Duan et al., 2013; Laila Roudsari et al., 2014; Yue et al., 2015
	Fibrin	Ye et al., 2000; Lieshout et al., 2006; Flanagan et al., 2007; Robinson and Tranquillo, 2009; Weber et al., 2014
	Collagen	Taylor et al., 2002; Flanagan et al., 2006; Tedder et al., 2008, 2010; Chen et al., 2013
	Elastin	Lu et al., 2004; Tedder et al., 2008; Chen et al., 2013; González De Torre et al., 2016

**FIGURE 1:**
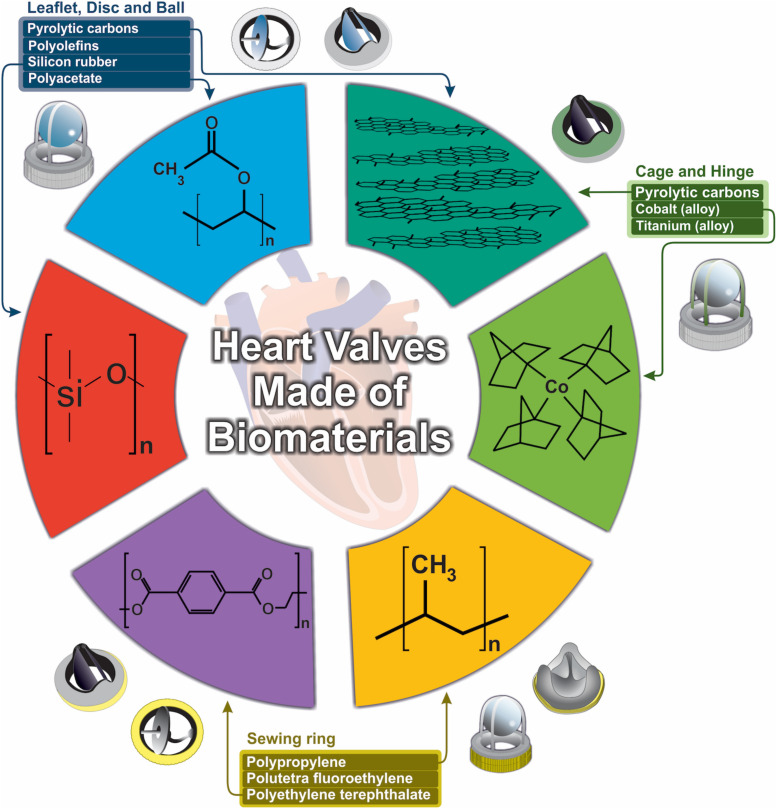
Design and development of biomaterials used in heart valves.

Fatigue and wear stress are two other major problems associated with valve prostheses failure (Klepetko et al., 1989). Generally, biomaterial’s fatigue is the result of body’s immune response to generated wear debris. This is why fatigue fracture and wear resistance of biomaterials should be addressed in PHV applications (Teoh, 2000). On the other hand, transvalvular pressure (following valve closure), is considered the heaviest burden on mechanical valves and can cause impact- and friction-wear stress (Legg et al., 2012). Since the first introduction of prosthetic heart valves, various models have been developed, each one made from different biomaterials and with specific geometry as well as mechanical and hemodynamic characteristics (Figure 1).

The main types of PHVs include ball in cage, tilting disc and bileaflet valves. The first successful prosthetic heart valve implantation (ball in cage valve, Figure 1), dates back to 1960s by Dr. Hufnagel. The Hufnagel valve consisted of an occluder ball, fabricated from methyl methacrylate (Plexiglas), restrained by a metal cage attached to a sewing ring, which was placed in the descending thoracic aorta instead of the heart itself. Plexiglas was later replaced by a silicone-coated hollow nylon poppet to reduce valve noise (Butany et al., 2002). The Starr-Edwards (SE) valve, which consisted of a methacrylate cage and a silicone elastomer rubber-ball, showed first promising results, since it had smaller size and could be placed inside the heart itself. Due to various complications associated with ball in cage valve (including aortic necrosis, pannus formation, paravalvular or paraprosthetic leakage, thrombosis, and infection), its production was discontinued in 2007. Most of these complications were related to non-optimal hemodynamic characteristics of these valves, including relatively large size of the occluder ball, which could damage blood cells in the passing blood flow. However, there has been an interesting case of a patient who had mitral stenosis and received SE valve transplant in 1974, with no major complications until 44 years after SE valve implantation (Hirji et al., 2018). Despite its disadvantages, the caged ball valve represented a new beginning in the area of biomaterials application in VHD management. Following the introduction of cardiopulmonary bypass technique in the early 1960’s, the next generation of PHVs were developed. Tilting disc (or mono-leaflet) valves are composed of a circular occluding disc attached to metal struts. This type of prosthetic valve allows more natural blood flow and less blood cell damage during blood passage through the valve, which results in lower occurrence of thromboembolism compared to the ball in cage valves (Raj, 2005). The tilting disc opens in an angle range of 60–80°, which provides two orifice areas with different sizes (Pibarot and Dumesnil, 2009). The first tilting disc valve was fabricated from Derlin polymer and introduced in the late 1960’s. The Derlin polymer was replaced by pyrolytic carbon (pyrolyte) in the later designs, since water absorption by the Derlin polymer resulted in valve disfiguration (Bjork, 1981). The thromboresistant feature of pyrolytic carbon further improved functionality of the tilting disc valves (Raj, 2005). However, one of the major complications associated with this type of mechanical heart valves is struts fracture due to fatigue.

Bileaflet valves consist of two semilunar leaflets attached to a rigid ring with the opening angle of 75–90°, resulting in three orifices with different sizes. Blood flow through these two leaflets, resembles natural heart valves, making bileaflet valves superior to other prosthetic valves (Raj, 2005). Today, bileaflet valves has become the most widely implanted valves due to their improved hemodynamic characteristics. Pyrolyte is the most commonly used biomaterial for production of the inner orifice and the leaflets of this type of prosthetic valve. However, despite its improved hemodynamics, presence of backflow is considered an important shortcoming.

Nowadays, commonly used materials in PHVs include elastomers (mainly polyurethanes and silicones) (Parfeev et al., 1983), titanium (Phillips, 2001; Zhou et al., 2015), pyrolytic carbon (Cao, 1996; Ely et al., 1998; Phillips, 2001) and metal alloys (Akins, 1979; Buchanan, 2005; Table 1).

Elastomers (so called “rubbers”) are commonly used in the biomedical field, mainly due to their ability to sustain deformation under stress conditions without being ruptured and resuming their original state upon removal of the stress. Elastomeric materials exist as natural and synthetic (including silicones and polyurethanes) forms (Yoda, 1998). Polyurethanes (PUs) have been the subject of extensive research in fabrication of PHVs (Braunwald et al., 1960). PUs are a large family of synthetic elastomers with diverse compositions and properties, which makes them one of the most biocompatible materials that currently exist. Despite these advantages, PU’s stability and susceptibility to biodegradation and calcification (leading to prosthesis premature failure), remains the major challenge in their application. Toxicity of biodegradation products of PUs is a concerning problem as well, since some of these products have been shown to be carcinogenic (Schoental, 1968; Cardy, 1979). The most commonly used PUs in valve prostheses include polyester urethane, polyether urethane (PEU) and polycarbonate urethane (Zdrahala and Zdrahala, 1999). A mechanical performance study by Bernacca et al. revealed that calcification in heart valves made of PEU was lower than the biological valves under the same conditions. Since PEU leaflets have lower thickness compared to bio-prosthetic valve leaflets, they are more susceptible to accumulation of calcified debris, which can be simply detached and released into the bloodstream. On the other hand, PEU valves have shown durability problems, as they have failed after 7.5 years following implantation (Bernacca et al., 1995, 1997a; Mackay et al., 1996). Further studies on the performance of PEU valves compared to urea-containing polyetherurethaneurea (PEUE) valves showed higher resistance and durability (up to 10 years) of PEUE compared to PEU valves (Bernacca et al., 1997a).

Surface modification is a useful strategy for manipulation of PU’s resistance to biodegradation, thrombogenesis and calcification. Alves et al. (2014) used 2 hydroxyethylmethacrylate (HEMA) with UV or argon plasma treatment for surface modification of Elastollan^®^ (a thermoplastic PU) and were able to improve surface hydrophilicity up to 30%, as well as enhanced antibacterial activity and hemocompatibility.

Bernacca et al. used heparin, taurine, 3-aminopropyltriethoxysilane and polyethylene oxide (PEO) to modify PEU and PEUE heart valves and to improved their fatigue resistance and durability (Bernacca and Wheatley, 1998). Many similar studies have reported the application of different materials to modify PU’s surfaces including sulfonated poly (ethylene oxide) (PEO) (Han et al., 2006) and poly (ethylene glycol) methacrylate (PEGMA) (Qiu et al., 1996). In one study by Stachelek et al. (2006) endothelial seeding of PU heart valve leaflets was accomplished by cholesterol modification of PU’s surface, promoting the resistance of the PU valve to thrombosis.

**FIGURE 2:**
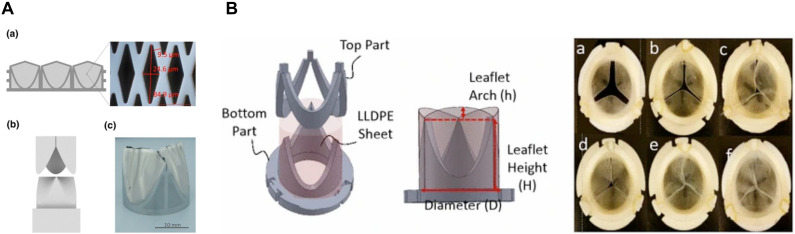
**(A)** Schematic micro-structured Nickle-Titanium (NiTi) thin film as a matrix scaffold for tissue engineered hybrid heart valves. (a) Formation of 3D valve leaflets by a steel mold. (b) Fabricated NiTi film valve leaflets. Reprinted with permission from Loger et al. (2016). Copyright (2016), Springer Nature. **(B)** Leaflets and stents assembly for fabricating heart valves (left), Top view of each six HVs with different arch profiles. Reprinted with permission from Yousefi et al. (2017). Copyright (2017), Springer Nature.

Flexibility of PU heart valves is obtained by using low modulus materials, however, low modulus PUs show poor durability due to high strain accumulation (Bernacca et al., 2002a). Since the first silicone rubbers used in PHVs fabrication tended to absorb lipids resulting in valve deterioration, they were later replaced by metal alloys. The strength, durability, and biocompatibility of metals have made titanium and stainless steel among the most common materials in PHVs fabrication (Van Putte et al., 2012; Loger et al., 2016; Figure 2A).

Pyrolytic carbon’s excellent features including its strength, clot forming prevention, and wear resistance as well as tissue- and blood-compatibility, make it an interesting biomaterial for fabricating PHVs (Bokros, 1989; Karp and Sand, 1993). Low-temperature isotropic (LTI) pyrolytic carbon is the most commonly used form of pyrolytic carbon in commercially available PHVs.

Blood-flow hemodynamics near the heart valve as well as the interactions of blood cells and platelets with valvular structures are among the most important determining factors for prosthetic valve’s success or failure. Since thrombosis and thromboembolism, are two major complications associated with PHVs, novel mechanical valves with improved hemodynamic characteristics are needed to be developed. The blood flow mechanics of each prosthetic heart valve design, will determine PHV’s performance. For example, in the ball in cage model, presence of a low-velocity recirculating blood flow with high shear stress downstream of the cage’s apex, which extends throughout the forward blood flow, has been identified. The turbulent shear stress in this region is high enough to activate platelets and trap them inside the recirculating blood flow, which in turn promotes platelet-platelet contacts and leads to thrombose formation at the apex region (Chandran et al., 1985; Wurzinger et al., 1985). As for the tilting-disc PHVs, two blood flows with different velocities emerge from the large and small orifices. Mixing of these two flow jets leads to generation of a major recirculating turbulent blood flow with high shear stress in the sinus region (Chandran et al., 1983). As mentioned before, presence of such turbulent flows promote platelet activation and blood cell damage. In bileaflet valves, turbulent blood flow with high shear stress generates due to high velocity gradient between the flow jets emerging from lateral and central orifices (Chandran et al., 2012). In addition to mentioned turbulent flows, blood leakage and regurgitating flow generation during valve closure, is also common in PHVs, in the locations of the gaps between the leaflets and at disk peripheries or in the hinge region. These regurgitating blood flows can enhance blood cell damage. Trileaflet valve’s hemodynamics closely resemble the native trileaflet aortic valve. However, incomplete closure of the leaflets during valve closure phase may lead to regurgitation (Leo et al., 2005, 2006).

In an interesting study by Yousefi et al. (2017), effect of leaflet’s geometry on PHV’s performance and hemodynamic features is investigated. They studied two geometric parameters including stent profile and leaflet arch length in six valve models (Figure 2B). They used different height to diameter rations (0.6, 0.7, and 0.88) and three arch heights to stent diameter ratios (0, 0.081, and 0.116) in their model designs. They found out that higher stent profile and presence of arches, reduce regurgitating flow generation, peak systole downstream velocity, subsequent shear stress and prevents loss of energy due to earlier reattachment of the forward blood flow (Yousefi et al., 2017).

**FIGURE 3:**
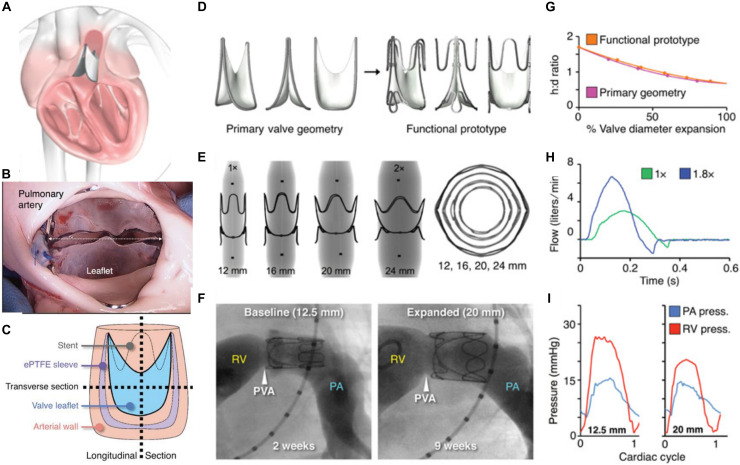
Expandable polytetrafluoroethylene (ePTFE) was used to fabricate the leaflets of the prototype, which were sewed to a stent made from stainless steel. **(A)** An arterial valve based on ePTFE located in the heart. **(B)** Photograph of implanted valve’s outflow surface. **(C)** The method of device sectioning after performing plastic embedding. **(D–F)** Valve expansion geometry for the primary valve geometry, X-ray images of laser-cut stainless steel functional valve prototypes being expanded via serial balloon dilation and representative right ventricular angiograms in a lamb. **(G–I)** Valve expansion geometry for the primary valve geometry and functional prototype, *in vitro* flow loop testing of functional prototype at two polar expansion states, and representative right ventricular and pulmonary artery pressures recorded at two states of valve expansion. Reprinted with permission from Hofferberth et al. (2020). Copyright (2020), American Association for the advancement of Science.

In a recent breakthrough, Hofferberth, and colleagues at the Harvard University introduced a geometrically adaptable bileaflet valve for children with congenital VHD, who need valve replacement. As mentioned earlier, currently the outcome of valve replacement in young children is not promising, since the patient will continually outgrow the replaced valve and need a new one. Since all the available prosthetic valves have fixed sizes and lack remodeling ability, developing novel PHVs with growth-accommodating ability is of particular importance. This group of researchers developed a biomimetic balloon-expandable PHV with adjustable size inspired from native human venous valves. They studied the performance and functionality of this new prosthetic valve via *in vivo* experiments in juvenile and adult ships, and used computational simulations to assess its stress-strain profile under physiological conditions. Three main geometrical factors were considered in the design of their prototypes including leaflet’s geometry, leaflets attachment and valve expansion geometries. A key parameter in geometrical consideration of their concept design was to fix the length of leaflet attachment, so that upon radial expansion, the valve’s height reduces. As a result, the valve shortens upon increasing the opening diameter, which fulfills valve coaptation without the need to increase leaflet’s surface. Expandable polytetrafluoroethylene (ePTFE) was used to fabricate the leaflets of the prototype, which were sewed to a stent made from stainless steel (Figure 3). *In vitro* experiments are performed in pediatric-specific hemodynamic conditions. In addition, they conducted computational modeling to study the stress-strain profile of the prototypes under physiological condition. Their results validated valve performance and durability. The adjustable dimensions of this valve allows it to be implanted at any age. Although, they used ePTFE for fabricating valve leaflets, the authors conclude that more research is needed to confirm that ePTFE is the ideal biomaterial for this purpose (Hofferberth et al., 2020).

Material selection is of great importance in designing the heart valves, since the biocompatibility and durability of the fabricated valves directly relates to the material’s performance in biological conditions. Selected polymers should possess proper affinity for endothelial cells attachment as well (Table 2; Kidane et al., 2009; Zarrintaj et al., 2018). Surface topology and chemical/physical features—including stiffness and hydrophilicity/hydrophobicity—determine the polymer’s biological response in the body’s physiological environment. As mentioned above, surface modification is a favorable technique to improve the surface interactions with biological milieu without deteriorating the bulk features. For example, plasma treatment, peptide conjugation, and cholesterol modifications have been shown to improve cellular attachment and activity. Such modifications can also enhance the endothelialization process, which is essential for reducing the immune responses and enhancing blood compatibility (Table 3).

**TABLE 2:** Different types of polymeric valves.

**Polymer**	**Properties**	**Disadvantage(s)**	**Performance**	**References**
PU	Good viscoelasticity	Rapid hydrolysis	Calcium deposition and biodegradation hinders its performance	Simmons et al., 2004
PEU	Resistance to hydrolysis	Susceptibility to oxidation		Christenson et al., 2004
PCU	Resistance to oxidation and hydrolysis	Susceptibility to calcification		Tang et al., 2001
PTFE	Good hemodynamics	Possible occurrence of thromboembolism, low resistance and high calcification and stiffening	Major complications	Nistal et al., 1990
Silicone	Good flexibility and biocompatibility	Low durability, distorted and thickened leaflets, tearing, thrombosis formation	Structural failure and impaired hemodynamic performance	Kiraly et al., 1982
PVA	Proper mechanical features	Not appropriate for dip-casting	Low elasticity	Jiang et al., 2004
PS-PIB-PS	High resistance to hydrolysis and oxidation	Platelet activation and thrombogenicity	Proper bio-stability	Gallocher et al., 2006
PDMS–PHMO PU	Proper mechanical properties; proper resistance to calcification and oxidation	Difficult processing	Proper bio-stability	Dabagh et al., 2005
POSS–PCU Nanocomposite	Proper resistance to oxidation, hydrolysis and calcification; high biocompatibility; anti-thrombogenicity		High bio-stability	Kannan et al., 2005

*PU, Polyurethane; PEU, polyether urethane; PCU, polycarbonate-urethane; PTEE, polytetrafluoroethylene; PVA, polyvinyl alcohol; PS-PIB-PS, poly (styrene–b–isobutylene–b–styrene); PDMS–PHMO PU, poly (dimethylsiloxane)/poly (hexamethylene oxide)-based polyurethane; POSS–PCU, polyhedral oligomeric silsesquioxane poly (carbonate-urea) urethane.*

**TABLE 3:** Modifications of polymeric valves.

**Modification**	**Advantages**	**Comments**	**References**
HEBP-bounded PU	Enhances resistivity to calcification	HEBP is used as an anti-calcification agents	Lamba, 2017
Cholesterol-modified PU	Improves endothelial cell adhesion	Enhances self-endothelialization by increasing cell affinity, decreases thrombosis formation and enzymatic degradation	Bergmeister et al., 2015
Fiber-reinforced SIBS	Ameliorates hemocompatibility	Improves mechanical features, stability and hemocompatibility	Hossainy et al., 2015
RGD incorporation	Promotes endothelial cell adhesion	Improves cell adhesion and endothelialization and hemocompatibility	Danilucci et al., 2019
PIII	Adjusts surface hydrophobicity	Enhances biocompatibility, weakens foreign body response	Chudinov et al., 2019
Nanotopographic surface	Enhances cellular activity	Might improve cell adhesion, growth, proliferation and differentiation	Zarrintaj et al., 2017
Incorporation of nanomaterials	Enhances mechanical features and durability	Nanoparticles can improve biocompatibility, resistance to calcification and stability	Nilforoushzadeh et al., 2018

*HEBP, 2-hydroxyethane bisphosphonic acid; PU, polyurethane; SIBS, Styrene Isoprene Butadiene; RGD, a tripeptide containing arginine, glycine and aspartic acid; PIII, Plasma immersion ion implantation.*

### Bio-Prosthetic Heart Valves (BPHVs)

Unlike PHVs, bio-prosthetic heart valves (or tissue valves), which are made partly or entirely from biological materials (derived from humans or animals), show good hemodynamic characteristics without necessary lifetime anticoagulation treatments. Natural biomaterials in BPHVs generally consist of ECM components or decellularized tissues such as arterial wall, pericardium, heart valve or small intestinal sub-mucosa (Schmidt and Baier, 2000; Hodde, 2002).

BPHVs can be classified as autografts (harvested from, and implanted into the same person), homografts or allografts (human heart valves removed post mortem), and xenografts. The Ross procedure (Switch procedure) is the autograft replacement of faulty aortic valve with patient’s own pulmonary valve. The pulmonary valve is then replaced with a homograft cadaveric pulmonary valve. This procedure was first introduced in 1967 and has been performed on young patients and children since (Ross, 1967). This durable BPHV will grow simultaneously with the patient’s heart, however, the procedure is complex and careful and proper young adult patient selection is necessary (Schaff, 2016). The growing shortage of available cardiac valves for transplantation has turned xenograft valves into valuable alternatives. Xenograft valves provide an unlimited source of valves prosthesis with different sizes, shapes, and anatomical configurations. Pericardial and porcine valves, made of cow’s pericardium and porcine aortic valve, respectively, are the most common types of xenograft BPHVs. The first successful xenograft valve transplant into a human was performed in 1965 (Binet et al., 1965). However, early attempts were accompanied with high rate of valve failure (nearly 60% failure in 1 year following the transplant), mainly due to host’s acute immune responses to xenograft tissue (Carpentier et al., 1969).

Different steps have been taken in order to increase xenograft valve’s stability and biocompatibility including; soluble protein removal by washing or electrolysis, sodium periodate denaturation of structural glycoproteins and mucopolysaccharides, neutralization by ethylene glycol and finally, glutaraldehyde treatment for cross-linking the remaining free amino groups of amino acids. Glutaraldehyde treatment is the most effective step in preserving and reducing the antigenicity of xenograft tissues (Manji et al., 2015). Despite increased functionality, biocompatibility, and mechanical strength of xenograft valves following glutaraldehyde fixation, occurrence of structural valvular deterioration (SVD) is still another major drawback (Manji et al., 2015). SVD is the result of host’s immune response to glutaraldehyde-treated tissue and subsequent calcification leading to valvular stenosis or regurgitation and eventual failure (Carpentier, 1989; Schoen and Levy, 2005; Manji et al., 2006). The rate of SVD occurrence is age dependent and is significantly higher in younger patients, mainly due to their robust immune system activity (Siddiqui et al., 2009) and accelerated rate of calcium metabolism (Simionescu, 2004). Nevertheless, glutaraldehyde-fixed BPHVs have limited durability of up to 15–20 years (Simionescu, 2004). Manufacturers have used different combinations of calcification-preventive agents in order to reduce SVD occurrence, including sodium dodecyl sulfate (SDS), α-amino oleic acid, toluidine blue by Medtronic (Minneapolis, MN, United States), ethanol by Epic (St. Jude Medical, Minneapolis, MN, United States) and ethanol with Tween-80 by Edwards Lifesciences Corporation, Santa Ana, CA, United States) (Simionescu, 2004).

Glutaraldehyde toxicity toward residual cells in xenograft valves is also associated with SVD, since dead resident cells cannot be removed from the tissue. These cells are not able to maintain calcium hemostasis and can initiate calcium nucleation leading to eventual SVD (Schoen and Levy, 2005).

Another issue that greatly affects BPHV’s durability is the expression of different antigenic epitopes (including α-Gal) in xenograft valves. The α-Gal epitope is not presented in the human body but it is preserved in non-primate mammals. However, the anti-Gal antibody constitutes 1% of immunoglobulins in the human body and is responsible for anti-gal reactivity and early rejection of xenograft BPHVs (Huai et al., 2016). Glutaraldehyde’s efficiency in inactivating or masking these epitopes is essential for successful BPHV preparation for clinical applications (Konakci et al., 2005). Naso et al. (2013) performed a quantitative evaluation of the number of α-Gal epitopes on seven commercially available glutaraldehyde-treated models of BPHVs. According to their results, the Epic valve was the only tested model that had completely masked α-Gal epitopes (Naso et al., 2013). They also suggested that α-Gal ELISA test can be applied as a quality control step for commercially available BPHVs. Decellularized tissues have also been prepared to minimize the possibility of immune rejection to xenograft valves. Decellularization mainly preserves ECM components to serve as a supporting scaffold in the process of damaged tissue repair (Byrne and Mcgregor, 2012). Decellularized tissues (such as decellularized aorta) brings major advantages including regeneration ability, ideal functional properties and biocompatibility (Wu et al., 2015).

More recently, the use of genetically modified animals expressing low levels of α-Gal epitopes have attracted researcher’s attention (Zeyland et al., 2014; Hryhorowicz et al., 2017). In this regard, Rahmani et al. (2019) have recently introduced a novel glutaraldehyde-fixed porcine pericardial BPHV obtained from transgenic pigs which showed excellent *in vitro* durability.

**FIGURE 4:**
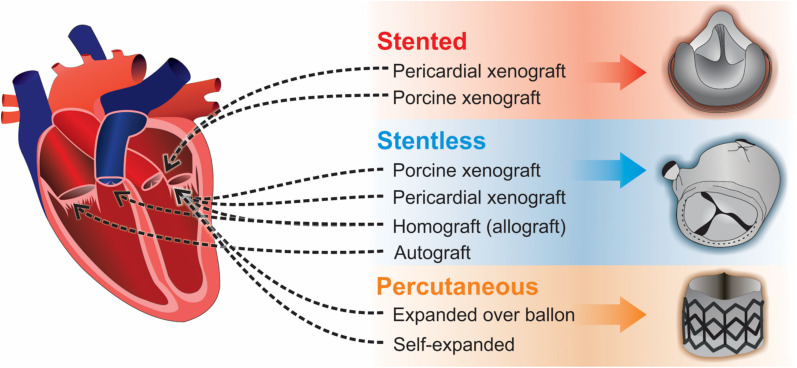
Three types of bioprosthetic valve replacements: stented, stentless, and percutaneous.

Three types of BPHV replacements exist: stented, stentless, and percutaneous (Figure 4). Stented BPHVs are generally made from glutaraldehyde-treated bovine pericardium or porcine aortic valve tissue sutured on a stent (polymeric or metal). Until early 2000s, bovine pericardium or porcine aortic valve tissue were the only options for fabrication of BPHVs. At that time, percutaneous transcatheter aortic valve implantation (TAVI) using equine pericardium as the biomaterial of valve leaflets, was introduced (Cribier et al., 2002). The first stented BPHV was developed in 1970s from glutaraldehyde-fixed bovine pericardium sewed on a flexible stent for obtaining a synchronous opening of the leaflets (Bartek et al., 1974). However, early SVD due to leaflet tearing within the stent was observed. Later BPHVs utilized thinner and more flexible stents in order to reduce valvular stress, allowing implantation of larger BPHVs. Patient-prosthesis mismatch (PPM) (due to small valvular effective orifice area) as well as stent-induced turbulent flow through the valve are important issues of current clinical practice. PPM puts patients at higher risks of postoperative mortality. Different modifications in stent architecture were proposed in order to overcome these problem, which eventually lead to development of stentless BPHVs in 1988 (David et al., 1988). Stentless BPHVs are made of porcine and bovine tissues without being sewed to a stent. They provide larger orifice area and reduced dimensions, both of which significantly affecting PPM. However, while the implantation of stented BPHVs is easier, especially through the small aortic root, surgical implantation of stentless valves is more difficult and time-consuming. In a recent study by Schaefer and colleagues, superior hemodynamic characteristics of stentless aortic valves in comparison to a stented aortic valve was confirmed, however, the rate of SVD occurrence and subsequent endocarditis was higher in patients who received stentless valves (Schaefer et al., 2018).

Percutaneous or trans-catheter valve replacement is the most advanced alternative approach to open heart surgery for patients with high risk of complications. Transcatheter endovascular delivery of BPHVs requires the BPHV to be crimped, which reduces its dimensions to up to 3-fold without rupturing the leaflets. Throughout these years, different issues have been reported that can potentially limit the application of percutaneous valves including paravalvular leakage, vascular, and neurological complications as well as complete atrioventricular blockage. The development of first percutaneous valve dates back to 1965, which was an umbrella-shaped valve placed on a catheter that was implanted into a dog (Andersen, 2009). An early percutaneous heart valve model was fabricated with a construct of folded wires to accommodate the valve. The prepared construct was then folded into a balloon shaped dilation catheter (Andersen et al., 1992). The next designed percutaneous valve was based on the ball and cage model (Pavcnik et al., 1992). After that, a platinum-iridium balloon-based stent was introduced by Boudjemline and Bonhoeffer (2002). Professor Alain Cribier performed the first percutaneous valve implantation in a human in 2002 (Cribier et al., 2002). Cribier valve was made of equine pericardium with a frame made of stainless steel. Introduction of a polyethylene terephthalate fabric cover to this percutaneous valve lead to development of the first Edwards SAPIEN valve model.

Based on a study published in 2009, aortic valve stent devices fabricated from stainless steel, cobalt chromium, nickel and titanium for percutaneous implantation, were characterized and evaluated by finite element analysis technique to assess the effect of biomaterial properties on stent performance under high blood pressure conditions. Their results indicated that titanium had the greatest impact on device performance, with maximum displacement and minimum stress levels (Kumar and Mathew, 2009).

### Prosthetic vs. Bio-Prosthetic Heart Valve Replacement

Current guidelines recommend using of MHVs for younger patients (<50 years of age) and BPHVs in patients over 70 years of age, and either type of these prosthetic valves can be used for patients in the range of 50–70 years old (Nishimura et al., 2017). The choice of valvular prosthesis is often made by evaluating the risk of re-operation and hemorrhage. In a recent study, Goldstone and colleagues evaluated the long-term post-operational complications including stroke, hemorrhage and mortality rate, in patients with severe valvular disease who underwent MHV or BPHV replacement, between 1996 and 2013 (Goldstone et al., 2017). According to their results, the risk of re-operation and mortality rate of patients who received MHVs was lower than those who received BPHVs. However, MHVs were associated with higher risk of hemorrhage and stroke. Similar results have been reported by Glaser et al. showing higher rate of long-term survival in patients who received MHVs than those who received BPHVs (Glaser et al., 2016). Other than that, several studies have reported higher risk of re-operation in patients aged 50–69 with BPHVs (Hammermeister et al., 2000; Oxenham et al., 2003; Chiang et al., 2014) which is mainly due to SVD and valve failure. Since valvular deterioration leading to re-operation limits the durability of BPHVs, this prosthesis is more suitable for older patients. Children will outgrow MHVs due to absent remodeling and will need re-operation. Younger patients can benefit more from BPHVs or TEHVs. Despite limited durability of BPHVs, application of these valve has increased compared to MHVs in older patients over the past few years, especially since the introduction of percutaneous transcatheter valve replacement. This approach is an excellent alternative for high-risk patients with similar safety outcomes as those of open-heart surgery (Makkar et al., 2020).

Children and young adults are not good candidates for mechanical heart valve replacement since they are at risk of complications due to long-term consumption of anti-coagulation medications and lack of implanted valve remodeling. On the other hand, they cannot fully benefit from advantages of BPHVs as well, due to early occurrence of SVD. Taken together, these patients are in need of suitable valvular prosthesis and extensive research in this area seems mandatory. α-Gal-free porcine pericardium obtained from transgenic pigs seems to be a promising biomaterial for increasing the durability of BPHVs, especially in younger patients. It should be noted that α-Gal is not the only antigenic epitope that can trigger immune responses in the recipient patients and evaluation of other xenogeneic antigenic epitopes might be essential for quality control assessment of BPHVs.

### Tissue-Engineered Heart Valves (TEHVs)

The ideal approach in valve replacement is to provide a viable valve capable of self-regeneration and growth, with greater life span and better biocompatibility. Tissue engineering, which is the use of biomaterials and engineering in combination with cells, offers a new generation of cardiac valves aiming to overcome the shortcomings of existing biological and mechanical heart valves (Rippel et al., 2012). To achieve this goal, different natural and synthetic biomaterials are investigated for fabrication of tissue-engineered heart valves (TEHV).

**FIGURE 5:**
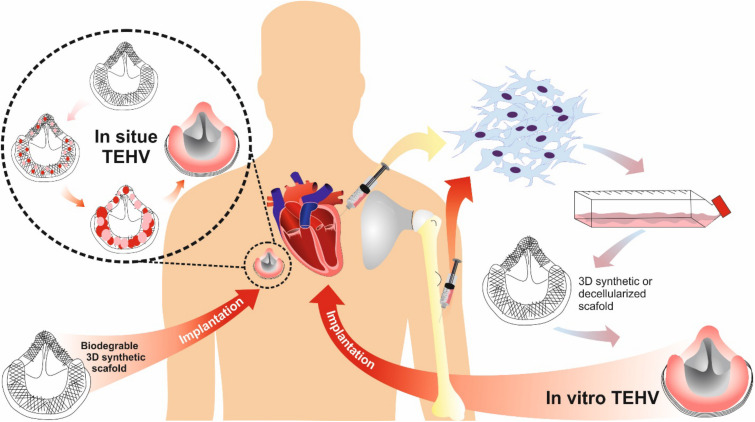
TEHV approaches comparison: *in situ* vs. *in vitro.*

**FIGURE 6:**
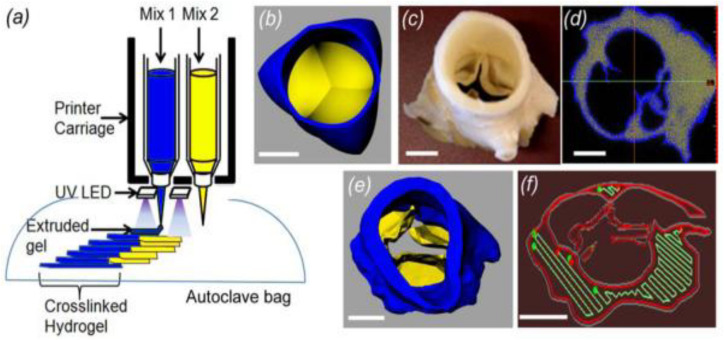
An engineered heterogeneous valve scaffold with poly-ethylene glycol-diacrylate (PEG-DA) hydrogels/alginate, supplemented with porcine aortic valve interstitial cells (PAVIC) by 3D-printing/photocrosslinking technique **(a)** 3D Printer setup of heterogeneous valve scaffolds. **(b–f)** Axisymmetric valve STL file of valve, micro-CT scan of aortic valve, the leaflet and root regions, the printable STL geometries of the threshold regions and the printing software sliced the geometries into layers and generated extrusion paths for each layer. Reprinted with permission from Hockaday et al. (2012). Copyright (2012), IOP science Publishing Ltd.

Two strategies for fabricating TEHVs include: *in vitro* and *in situ* valve heart tissue engineering (Figure 5). The *in vitro* approach is based on the *ex vivo* formation of TEHV, which includes harvesting of autologous cells from the patient, cell seeding on a 3D scaffold, *in vitro* tissue generation and finally, implantation of the generated tissue into the same patient. Hockaday et al. (2012) introduced an engineered heterogeneous valve scaffold made from poly-ethylene-glycol-diacrylate (PEG-DA) hydrogel and alginate, supplemented with porcine aortic valve interstitial cells (PAVIC), fabricated by 3D-printing/photo-crosslinking technique (Figure 6). Their results demonstrated that this 3D scaffold supports cell engraftment and provides acceptable dynamics and mechanical heterogeneity (Hockaday et al., 2012). The *in situ* approach, on the other hand, involves using biological or polymeric heart valve scaffolds, which are seeded with patient’s autologous cells and upon implantation undergo subsequent cell attraction and tissue remodeling (Weber et al., 2012).

As mentioned earlier, endothelial and interstitial cells are responsible for forming and maintaining valvular integrity (Brody and Pandit, 2007). Since VECs show anti-thrombogenic properties, they are being used for surface cell lining, while VICs produce ECM components (Hoerstrup et al., 2000). Currently, patient-derived endothelial and interstitial cells are the main cell sources for TEHVs fabrication. The cell isolation process involves sacrificing healthy donor vascular tissues, while the isolated cell’s function could also be affected by risk factors including diabetes or atherosclerosis (De Vriese et al., 2000). To avoid these complications, stem cells from different sources are available, including non-hematopoietic bone marrow-derived stem cells (mesenchymal and mononuclear stem cells) (Hoerstrup et al., 2002; Sutherland et al., 2005; Schmidt et al., 2010; Weber et al., 2011), blood-derived endothelial progenitor cells (Schmidt et al., 2010), mesenchymal stem cells derived from adipose tissue (Colazzo et al., 2010), amniotic fluid (Schmidt et al., 2007), chorionic villi (Schmidt et al., 2006a) and the umbilical cord (Schmidt et al., 2006b; Sodian et al., 2010).

The scaffold provides a 3D structure for cellular attachment and tissue growth. Structural characteristics of the scaffold (including pore size, surface biochemistry and topology, biocompatibility and biodegradation as well as mechanical strength) can pre-determine TEHV’s performance (Nie et al., 2014). In general, two types of scaffolds exist for fabrication of TEHVs including decellularized ECM and synthetic biomaterials. Decellularized scaffolds are the most promising choices for TEHV fabrication, which can have allogeneic and xenogeneic sources or be tissue-engineered with intact ECM. Shortage of decellularized allogeneic valves limits their availability. In this regard, decellularized xenogeneic valves are the best alternatives. In hybrid scaffolds, an inorganic mesh with high mechanical resistance is exposed to valvular tissues. Loger et al. designed a 3D Nickle-Titanium (NiTi) hybrid scaffold for engineering thin, durable, and efficient valve leaflets for transcatheter implantation. Their fabrication process involved magnetron sputtering, lithography, wet etching, and a final step for shape setting the scaffold. The scaffold was exposed to cardiac smooth muscle cells. The performance of fabricated leaflets was studied under pulsatile condition *in vitro* and was compared to a porcine valve as a reference. Their results showed higher effective opening area, lower systolic transvalvular pressure due to increased cardiac output and low flow resistance. They suggest further design optimization with increased duration of cell seeding process as well as addition of cardiac fibroblasts and VECs could improve the durability of their NiTi thin film leaflets (Loger et al., 2016; Figure 2A).

Different techniques used for decellularization can be classified as physical, chemical (or enzymatic) or the combination of both. Each technique consists of the following steps: cell lysis and removal, genetic material removal (for lowering the immunogenicity) and preserving the composition and structure of the ECM (Gilpin and Yang, 2017). Chemical decellularization is usually performed by using surfactants (SDS and TritonX-100, Tween 20, TnBP, CHAPS), acids, bases, and enzymes (pancreatin and trypsin) (Nie et al., 2014). Despite their wide application, toxicity of these chemical agents, as well as damage to the structural proteins of the EMC are concerning. Physical techniques including freeze-thaw, ultrasonication, high hydrodynamic pressure (HHP) and supercritical carbon dioxide (CO_2_) treatment are alternative approaches. Due to specific advantages and disadvantages of each technique, combinatorial treatments may be performed in order to achieve the best results. For example, physical approaches are more successful in maintaining intact ECM, while they are unable to eliminate tissue’s immunogenicity. Chemical approaches on the other hand are not ideal for removing cellular debris. Combining these two approaches can yield an intact ECM with no debris. Ramm and colleagues in 2019 studied decellularized porcine pulmonary heart valves (dpPHVs), implanted in sheep. They used nine different techniques for generation of these dpPHVs. They evaluated the amount of residual SDS as well as DNA, GAGs and hydroxyproline content of dpPHVs and observed that addition of trypsin to Triton X-100 in the decellularization process results in efficient removal of cellular debris, while preserving the ECM. Trypsin- and Triton X-100-treated pdPHV’s performance was comparable to allogeneic decellularized valves. For DNA and N-linked glycans removal, they observed that the combination of PNGase F and DNase I with Triton X-100 and SDS improved the efficacy. However, the combination of trypsin and Triton X-100 resulted in lowest immunogenicity (Ramm et al., 2020). Among different chemical agents used for decellularization, SDS is more common, since it can almost completely remove caridac valve cells, while preserving the ECM. However, possible toxicity of these decellularized scaffolds due to leakage of the remained SDS within the valve’s tissue is a matter of concern. It is reported that following several washing steps, even when small SDS concentrations are used (0.01%), SDS is still present in the tissue (Naso and Gandaglia, 2018). Another limitation in the application of SDS is insoluble elastin degradation following SDS exposure, which negatively affects ECM’s tensile strength (Jordan et al., 1974).

The issue of decellularized valve’s immunogenicity should also be addressed. Hyperacute, acute and chronic rejection may occur following xenogeneic decellularized tissue implantation due to presence of xenoantigens. The process of decellularization can increase tissue’s immunogenicity, itself. For example, cardiovascular ECM is reach in hyaluronan (HA) polymers (Ding et al., 2019). Anti-inflammatory actions of HA provides cellular protection via inhibiting phagocytosis by macrophages, neutrophils and monocytes (Delmage et al., 1986). Despite their protective role, HA fragments (which are produced via HA filaments degradation by released proteases during the first step of decellularization), can be pro-inflammatory and lead to acute rejection of the implanted valve (Naso and Gandaglia, 2018). One of the most effective strategies for lowering decellularized tissue’s immunogenicity is targeted antigen removal of well-known xenoantigens (for example; targeted removal of α-gal by α-galactosidases) (Nam et al., 2012). Unknown xenogeneic antigens removal is performed through solubilization-based techniques. This strategy is based on the fact that all tissue components need to be solubilized in order to be removed from the ECM. The solubilization-based approaches do not require identification of specific graft’s antigens and are based on the xenoantigen’s solubility in a common antigen removal buffer (Wong and Griffiths, 2014). Another issue regarding the safety of decellularized cardiac valves is complete removal of cell debris. Although decellularized tissue is considered the most suitable substrate for fabrication of TEHVs, in terms of 3D structure, composition, and function, its limited availability and the risk of possible immunogenicity restrict its application (Schenke-Layland et al., 2003).

Natural polymesr including collagen, elastin (Chen et al., 2013), polyhydroxyalkanoates (PHA) (Sodian et al., 2000), polyethylene glycol (PEG) (Zhang et al., 2015), PEG-poly lactic acid (PEG-PLA) (Hinderer et al., 2014), and poly glycerol sebacate (PGS)-Polycaprolactone (PCL) (Masoumi et al., 2014), can be used as alternatives for decellularized scaffolds. One of the main challenges of long-term application of polymer-based 3D scaffolds is calcification or tearing. Several strategies have been used to prevent calcification, including local administration of bisphosphonates (Levy et al., 1985; Golomb et al., 1986, 1987), bisphosphonate-bound PUs (Alferiev et al., 2001) and ethanol pre-treatment of glutaraldehyde cross-linked bio-prosthetic heart valves (Vyavahare et al., 1997).

Ozaki et al. (2011) introduced a novel aortic valve repair procedure using glutaraldehyde-treated autologous pericardium, which could be an alternative for aortic valve replacement in older patients who are candidate for aortic valve replacement. As mentioned earlier, bioprosthetic valve transplantation is the preferred option for older patients, and valve repair is not routinely performed in these cases. Since the performance of biological valves is far from ideal, repairing patient’s own aortic valve becomes an interesting alternative. In Ozaki procedure, patient’s pericardium is harvested and treated with glutaraldehyde prior to be trimmed based on the measured value of the distance between each commissures. Treatment with glutaraldehyde can increase pericardium’s resistance and maintain tissue’s intrinsic elasticity. Trimmed glutaraldehyde-treated autologous pericardium is sewed into the native aortic valve’s annulus and root, following removal of the diseased valve leaflets. The Ozaki procedure is applicable to different types of aortic valve diseases and can be performed by using bovine or patient’s own pericardium (Ozaki et al., 2011). Although this procedure was primarily developed for treatment of aortic valve diseases in older patients, it has now become an attractive strategy for children with congenital valve disease since the new valve leaflets can expand along with patient’s heart growth.

Once the scaffold is ready and seeded, the new construct is placed inside a bioreactor in order to get adapted to the physiological biochemical and mechanical conditions. This process prepares the TEHV for implantation into the patient’s body. Bioreactors may also be used to study cellular functionality, cell-cell and cell-ECM interactions as well as cellular responses to physiological conditions of the valve prior to implantation. Various bioreactor designs have been developed for this purpose including cell-stretching bioreactors (Engelmayr et al., 2008; Syedain and Tranquillo, 2009), dynamic flexure bioreactors (Engelmayr et al., 2003), and flow bioreactors (Jockenhoevel et al., 2002; Ramaswamy et al., 2014). In addition, different commercial models of bioreactors are also available. By studying cellular and ECM responses to the physiological conditions of the target valve’s environment in the human heart prior to TEHV implantation, a wealth of information would be obtained, which could be very useful in choosing the proper cell types and biomaterials.

## Future Challenges and Directions

Despite numerous attempts over the past decades in the area of design and development of biomaterial-based cardiac valves, this task remains extremely challenging. This is partly due to the difficulty of replicating biomechanical features of native heart valves, since cardiac valves are not merely passive systems and undergo constant on-demand adjustment and remodeling. One of the main purposes of studying valve biomechanics is to understand the mechanisms by which the biomechanical forces are transmitted to valvular cells and the ECM. This can be achieved by using mathematical modeling and computer simulations. The level of complexity of valvular micro and macrostructure should be considered in material selection for prosthetic heart valves. In this regard, more research toward better understanding of valvular biomechanics via accurate simulations and mathematical modeling can lead to significant advancements in the performance of designed valves. This task requires a thorough understanding of the biomechanics of healthy and diseased valvular tissue, and with help of state-of-the-art techniques (such as nanotechnology), biomaterials with adjustable characteristics can be obtained.

Both medical professionals and researchers strive to provide durable hemocompatible, anticoagulant-independent and thrombus-free heart valves with the ability of remodeling. While these attempts are not far from becoming the standard routine in clinical practice, novel biomaterials with adjustable properties and mechanized industrial processing techniques are currently under research, since biomaterial’s properties, cost and availability are among the most important determining factors in material selection for biomaterial-based valve prosthesis.

Biomaterials have shown great potential in management of CVD. Biocompatibility and biodegradation rates of most common biomaterials have been widely studied by various research groups. The optimum biomaterial-based heart valve should possess combined advantages of MHVs and BPHVs. It seems that the future of biomaterials-based CVD management is headed toward TEHVs. However, the optimum combination of cells and biomaterials for promotion of tissue regeneration is yet to be identified.

Another concern that needs to be addressed, is minimizing the immunogenicity of applied biomaterials or tissue constructs, as they may trigger immune responses which have negative impact on their efficacy and performance.

Despite these challenges, research in the field of biomaterials and tissue engineering for CVD treatment has achieved significant progress. However, the gold standard approach would provide the ability of using biomaterial scaffolds in combination with suitable cell types with good functionality, biocompatibility, and durability, with the aim of providing partial or whole heart regenerational capacity. Achieving this goal will ultimately eliminate the need for heart transplant in patients with heart failure, manage disease states in which irreversible functional loss has occurred and result in improved patient’s quality of life.

## Author Contributions

ZI, LT, and HD performed the main conceptual ideas and proof outline. BT, HD, and LG wrote the manuscript with support MR, MJ, and PZ. NZ, MJ, MM, and ZI designed the figures. LT supervised the manuscript and final approval of the version to be published. All authors contributed to the article and approved the submitted version.

## Conflict of Interest

The authors declare that the research was conducted in the absence of any commercial or financial relationships that could be construed as a potential conflict of interest.
